# Neuropsychiatric and behavioral markers of dementia risk

**DOI:** 10.1002/alz.089506

**Published:** 2025-01-03

**Authors:** Zahinoor Ismail

**Affiliations:** ^1^ Hotchkiss Brain Institute, University of Calgary, Calgary, AB Canada

## Abstract

A critical need exists to find simpler approaches for early detection of Alzheimer disease (AD) and related dementias. These approaches must increase access to preventative interventions and treatments, both pharmacological and non‐pharmacological, for people in preclinical and prodromal stages of disease. This need has been amplified by the emergence of disease modifying therapies (DMTs), which require biological confirmation of amyloid‐ß positivity. Confirmation methods include positron emission tomography (PET) tracers, lumbar puncture for cerebrospinal fluid analysis, and phlebotomy for blood assays. However, given the expense, invasiveness, and lack of availability to all, these methods are not suitable for larger scale screening or case detection. Finding higher‐risk persons, enriched for biomarker positivity, can help with case detection. Digital biomarkers, delivered at scale, may have a role. Conventionally, the at‐risk group is identified based on cognitive symptoms. An underappreciated approach to help with detection of AD is to incorporate neuropsychiatric symptoms (NPS) into screening or assessment. Historically, specificity has been a limitation, with NPS manifesting due to other, non‐AD aetiologies. More recently, the construct of Mild Behavioral Impairment (MBI) has been developed to improve specificity for AD detection and prognostication when using NPS. MBI leverages risk associated with later‐life emergent and persistent NPS to improve specificity. For example, in persons with Subjective Cognitive Decline or with Mild Cognitive Impairment, stratifying by MBI status selects a subgroup with higher rates of incident cognitive decline and dementia. Even more advantageous has been the utility of MBI to predict MCI and dementia in cognitively unimpaired participants, for whom cognitive symptoms cannot easily be used to detect risk. More recently, evidence has emerged supporting the role of MBI for detection of persons with elevated AD biomarker levels, with greater specificity than NPS not meeting MBI criteria. These findings extend the epidemiological data and highlight MBI as a potential tool for sample enrichment. With MBI validated for digital delivery, there is potential to improve screening and case detection at scale. In this session, we will review the MBI construct, the role of MBI in dementia prognostication, and the most current data linking MBI to amyloid, tau, and neurodegeneration.

